# A dominant *RAD51C* pathogenic splicing variant predisposes to breast and ovarian cancer in the Newfoundland population due to founder effect

**DOI:** 10.1002/mgg3.1070

**Published:** 2019-11-28

**Authors:** Lesa M. Dawson, Kerri N. Smith, Salem Werdyani, Robyn Ndikumana, Cindy Penney, Louisa L. Wiede, Kendra L. Smith, Justin A. Pater, Andrée MacMillan, Jane Green, Sheila Drover, Terry‐Lynn Young, Darren D. O’Rielly

**Affiliations:** ^1^ Faculty of Medicine Memorial University of Newfoundland St. John’s NL Canada; ^2^ Eastern Health Authority St. John’s NL Canada; ^3^ Centre for Translational Genomics St. John’s NL Canada

**Keywords:** founder variant, hereditary breast and ovarian cancer, RAD51C, splicing, variant of uncertain significance

## Abstract

**Background:**

RAD51C is important in DNA repair and individuals with pathogenic *RAD51C* variants have increased risk of hereditary breast and ovarian cancer syndrome (HBOC), an autosomal dominant genetic predisposition to early onset breast and/or ovarian cancer.

**Methods:**

Five female HBOC probands sequenced negative for moderate‐ and high‐risk genes but shared a recurrent variant of uncertain significance in *RAD51C* (NM_058216.3: c.571 + 4A > G). Participant recruitment was followed by haplotype and case/control analyses, RNA splicing analysis, gene and protein expression assays, and Sanger sequencing of tumors.

**Results:**

The *RAD51C* c.571 + 4A > G variant segregates with HBOC, with heterozygotes sharing a 5.07 Mbp haplotype. *RAD51C* c.571 + 4A > G is increased ~52‐fold in the Newfoundland population compared with the general Caucasian population and positive population controls share disease‐associated alleles, providing evidence of a founder effect. Splicing analysis confirmed in silico predictions that *RAD51C* c.571 + 4A > G causes exon 3 skipping, creating an immediate premature termination codon. Gene and protein expression were significantly reduced in a *RAD51C* c.571 + 4G > A heterozygote compared with a wild‐type relative. Sanger sequencing of tumors from two probands indicates loss‐of‐heterozygosity, suggesting loss of function.

**Conclusion:**

The *RAD51C* c.571 + 4A > G variant affects mRNA splicing and should be re‐classified as pathogenic according to American College of Medical Genetics and Genomics guidelines.

## Graphical abstract

RAD51C is important in DNA repair and carriers of pathogenic RAD51C variants have increased risk of hereditary breast and ovarian cancer syndrome (HBOC), an autosomal dominant genetic predisposition to early onset breast and/or ovarian cancer. This study identified five female HBOC probands that shared a recurrent variant of uncertain significance in RAD51C (NM_058216.3: c.571 + 4A > G). Our analyses demonstrated that the RAD51C c.571 + 4A > G variant affects mRNA splicing and should be re‐classified as pathogenic according to American College of Medical Genetics and Genomics guidelines.

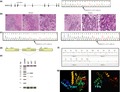

## 1 INTRODUCTION

Hereditary breast and ovarian cancer syndrome (HBOC) is an autosomal dominant genetic predisposition to early onset breast cancer (BC) and/or ovarian cancer (OC). Approximately 25% of HBOC cases are attributed to pathogenic variants in *BRCA1* (OMIM: 113705) and *BRCA*2 (OMIM: 600185), which encode proteins involved in homologous recombination (HR) DNA repair (Nielsen, van Overeem Hansen, & Sorensen, 2016). At least two dozen genes have been associated with HBOC (Nielsen et al., 2016), encoding proteins involved in HR that manifest in a “BRCAness phenotype” including younger age at diagnosis, primarily ovarian high‐grade serous carcinoma (HGSC) histotype, and a family history of HBOC (Lord & Ashworth, 2016). Despite increased risk of OC in females from HBOC families, HR‐deficient tumors are highly amenable to platinum‐based chemotherapies and inhibitors of HR repair, leading to improved health outcomes (Capoluongo et al., 2017). Multigene sequencing panels have become standard of care for clinical HBOC testing (Nielsen et al., 2016); however, an increase in variants of uncertain significance (VUS) poses a significant challenge for patient management. Familial investigations, functional studies, and population‐level data with accurate phenotyping will improve variant classification and reduce uncertainties in clinical interpretation.

*RAD51C* (OMIM: 602774) was first associated with HBOC families in 2010 (Meindl et al., 2010). RAD51C interacts with other RAD51 paralogs, BRCA1/2 and their associated proteins as critical components of HR and DNA repair. Specifically, RAD51C is involved in both early stages of HR repair by binding with the CX3 complex, and in late stages by binding with Holliday Junction substrates, promoting branch migration and resolution (Chun, Buechelmaier, & Powell, 2013). Multiple studies have identified an association between *RAD51C* pathogenic variants and OC, or families with BC and OC in different populations (Blanco et al., 2014; Clague et al., 2011; Eoh et al., 2018; Jonson et al., 2016; Loveday et al., 2012; Lu et al., 2018; Meindl et al., 2010; Neidhardt et al., 2017; Osorio et al., 2012; Pang et al., 2011; Pelttari et al., 2011, 2018; Romero et al., 2011; Sanchez‐Bermudez et al., 2018; Schnurbein et al., 2013; Song et al., 2015; Sung et al., 2017; Thompson et al., 2012; Vuorela et al., 2011). Collectively, *RAD51C* pathogenic variants have an overall prevalence of 0.84% in HBOC families as previously reviewed (Sopik, Akbari, & Narod, 2015). Indeed, there is strong evidence that *RAD51C* pathogenic variants are associated with an increased risk of OC (Blanco et al., 2014; Clague et al., 2011; Eoh et al., 2018; Jonson et al., 2016; Loveday et al., 2012; Lu et al., 2018; Meindl et al., 2010; Neidhardt et al., 2017; Osorio et al., 2012; Pang et al., 2011; Pelttari et al., 2011, 2018; Romero et al., 2011; Sanchez‐Bermudez et al., 2018; Schnurbein et al., 2013; Song et al., 2015; Sung et al., 2017; Thompson et al., 2012; Vuorela et al., 2011); however, their contribution to BC remains uncertain. Moreover, individuals with pathogenic *RAD51C* variants are not candidates for increased breast surveillance or prophylactic surgical interventions because there is no indication of an elevated risk of BC (Couch et al., 2017; Sopik et al., 2015), with the exception of triple negative breast cancer (Shimelis et al., 2018).

Newfoundland and Labrador (NL), a recognized founder population of English and Irish descent (Zhai et al., 2016), has the highest incidence and mortality rate for BC and one of the highest incidence and mortality rates for OC in Canada (Canadian Cancer Statistics, 2017). Recurrent variants representing a founder effect generate considerable interest as they facilitate the study of penetrance and prevalence, although prevalence may not be generalizable due to genetic drift. The genetic structure of the NL founder population and the availability of extended families with large sibships, combined with a centralized cancer program, makes NL an ideal population to discern disease‐causing variants. Previous studies have shown founder effects with colorectal cancer (Green et al., 1994) and multiple endocrine neoplasia type 1 (Olufemi et al., 1998; Petty et al., 1994) in NL. Given that the association of *RAD51C* with HBOC is relatively new and the frequency of pathogenic variants relatively low, studying *RAD51C* variants in a genetic isolate is a powerful method to determine clinical significance of a VUS and its natural history over time. We report on five female probands (two with BC and three with OC) and their extended families in which we identified a VUS in *RAD51C* (NM_058216.3: c.571 + 4A > G; rs587780257). Detailed clinical evaluations, pedigree construction, haplotype analysis, and functional studies revealed a dominant‐acting *RAD51C* c.571 + 4A > G splicing variant residing on a shared HBOC‐associated haplotype that increases the risk for BC in addition to OC.

## 2 MATERIALS AND METHODS

### 2.1 Ethical compliance

Ethical approval for this study was granted through the Human Research Ethics Authority of NL (protocols #2016.1914 and #2018.0391) and the Research Proposal Approval Committee (Eastern Health Authority). Informed written consent and permission to access medical records were obtained from all participants.

### 2.2 Study participants and family pedigrees

**Figure 1:**
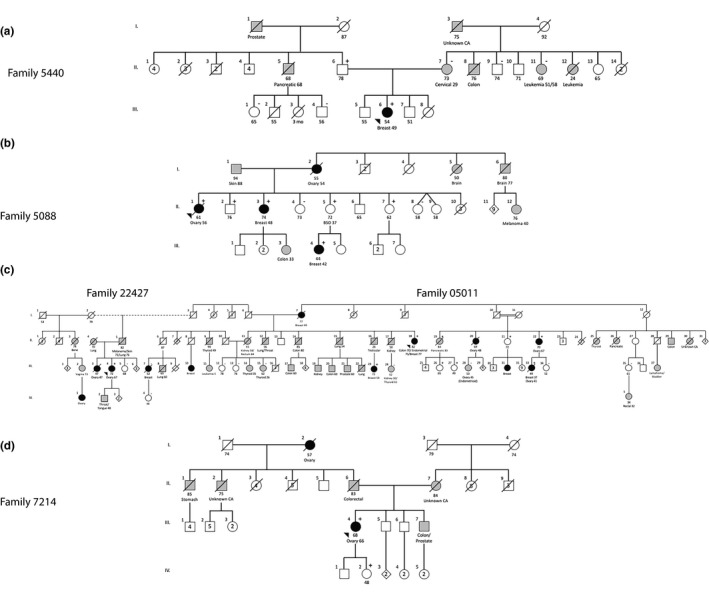
*RAD51C* c.571 + 4A > G segregates with breast and/or ovarian cancer in five multiplex families (a–d) that tested negative for variants in *BRCA1*, *BRCA2*, and other high and moderate cancer susceptibility genes. Family pedigree number, age at cancer diagnosis and current age of individuals are indicated. Black shade: diagnosis of breast and/or ovarian cancer; gray shade: diagnosis with cancer unrelated to HBOC; white shade: no cancer diagnosis; arrows: probands; slash: deceased; +: *RAD51C* c.571 + 4A > G heterozygote; −: *RAD51C* c.571 + 4 wild‐type

Seventy subjects were recruited from the Provincial Medical Genetics Program and the NL HBOC Study (a population‐based study to determine the burden of inherited BC and OC in the NL population). Five probands underwent family history and pedigree evaluations and clinical risk was assessed according to formal criteria (Table S1). Following routine clinical assessment, probands were offered genetic testing as per local clinical care standard (Figure 1). Two probands belong to an extended pedigree related by marriage; however, two separate family numbers (22427 and 05011) were retained because the probands are not blood relatives (Figure 1). Health records of extended relatives were reviewed for relevant cancer diagnoses and extensive genealogical investigation, including community of origin for pedigree founders, was obtained from family interviews, census data, and church records.

### 2.3 Multigene sequencing

A panel of moderate‐ and high‐risk cancer susceptibility genes was performed at external facilities using the OncoGeneDx Custom Panel (GeneDX), the Common Hereditary Cancers Panel (Invitae) or the Ovarian Cancer Focus Panel (Fulgent Genetics) with a minimum 50× depth of coverage (Table S2). The index case (proband of family 5440) screened negative for pathogenic variants or likely pathogenic variants in the multigene panel (Figure 1a). This was followed by performing whole‐exome sequencing and genome‐wide microarray analysis at the Centre for Translational Genomics (St. John's, NL, Canada) to detect other potential disease‐causing variants (Data S1). Four additional probands, including three with HGSC and one with BC also screened negative for pathogenic variants or likely pathogenic variants in moderate‐ and high‐risk cancer susceptibility genes (Figure 1). A recurrent VUS in *RAD51C* (NM_058216.3: c.571 + 4A > G; rs587780257) was identified in five unrelated probands. One proband (family 7214) also carried a likely pathogenic variant in *CHEK2* (OMIM: 604373; NM_007194.4: c.470T > C; rs17879961). Given that *CHEK2* is a low penetrance cancer susceptibility gene (Zannini, Delia, & Buscemi, 2014) and the lack of a more severe phenotype (e.g. younger age of onset) in that proband, the *CHEK2* variant was not further investigated.

### 2.4 Variant validation and family cascade genotyping analysis

For genomic analysis, EDTA‐anticoagulated whole blood was collected from all participants, and DNA was extracted using a simple salting‐out method (Miller, Dykes, & Polesky, 1988). Variants of interest identified in the five probands were validated by Sanger sequencing using custom‐designed primers (Table S3) and Platinum *Taq* DNA Polymerase (Invitrogen, #10966034). Excess nucleotides and primers were removed using a standard ExoSAP‐IT (Applied Biosystems, #78201) protocol and amplicons were sequenced using a BigDye Terminator v3.1 Cycle Sequencing Kit (Applied Biosystems, #4337455). Amplified products were purified using a BigDye XTerminator Purification Kit (Applied Biosystems, #4376486) and sequenced on a 3130*xl* Genetic Analyzer (Applied Biosystems). DNA sequences were analyzed using Mutation Surveyor Software v5.0.0 (SoftGenetics LLC). Extended family members were genotyped using a TaqMan assay specific for the *RAD51C* c.571 + 4A > G variant (Applied Biosystems, Assay ID C_341775427_10; Table S3) using a 7900HT Fast Real‐Time PCR System (Applied Biosystems). Data were analyzed using TaqMan Genotyper Software v1.4 (Applied Biosystems). The TaqMan assay was used to screen the remaining 65 cancer cases (58 OC; 7 BC), as well as 880 ethnically matched NL population controls for the *RAD51C* c.571 + 4A > G variant.

### 2.5 Haplotype analysis

Polymorphic microsatellite markers (*D17S666*, *D17S1853*, *D17S1606*, *D17S1161*, *D17S1604*, *D17S923*, *D17S808*, and *D17S794*) flanking the recurrent *RAD51C* c.571 + 4A > G variant were genotyped in available relatives (Table S3), alleles were scored with GeneMapper Software v5.0 (Applied Biosystems), and haplotypes were constructed according to standard procedures (Abdelfatah et al., 2013). Specific alleles residing on the disease‐associated haplotype were compared with genotypes of a positive singleton case and population controls. Estimated variant age was calculated using standard procedures (Machado et al., 2007), with genetic distances inferred from the deCODE genetic map (Halldorsson et al., 2019) (Data S1).

### 2.6 Splicing analysis

In silico splicing analyses were performed using a custom variant annotation pipeline and Alamut Visual v2.11 (Interactive Biosoftware) and Phyre2 was used for protein modeling (Kelley, Mezulis, Yates, Wass, & Sternberg, 2015). To experimentally confirm splicing effects, patient‐derived peripheral blood mononuclear cells (PBMCs) were separated by density gradient centrifugation using Ficoll‐Paque PLUS (GE Healthcare, #17144002), washed with HBSS (Gibco, #14170112) and re‐suspended in RPMI 1640 Medium (Gibco, #11875093) supplemented with 10% heat inactivated fetal bovine serum (Gibco, #12483020), 100 U/ml‐100 µg/ml penicillin‐streptomycin (Gibco, #15070063), 10 mM HEPES (Gibco, #15630080), 1 mM sodium pyruvate (Gibco, #11360070), 2 mM GlutaMAX‐I (Gibco, #35050061), and 1.4 µg/ml antibiotic‐antimycotic (Gibco, #15240062). Lymphoblastoid B cell lines (BCL) were generated from a proband and a wild‐type relative by Epstein‐Barr transformation of PBMCs and cultured as previously described (Mason, Bowmer, Howley, & Grant, 2005).

Experimental splicing analysis was performed by amplifying cDNA from BCL with specific primers across *RAD51C* exons 2 and 4 (Table S3) using Platinum *Taq* DNA Polymerase (Invitrogen, #10966034). Amplified PCR products were visualized using a D1000 ScreenTape (Agilent Technologies, #5067–5582) on an Agilent 2200 TapeStation System (Agilent Technologies) and run on a 1% agarose gel. Bands were excised and purified using a QIAquick Gel Extraction Kit (Qiagen, #28704) and sequenced. *RAD51C* transcript splicing was also analyzed by TOPO TA cloning (Data S1). The effect of the *RAD51C* variant on protein weight was predicted using a publicly available online resource (https://www.bioinformatics.org/sms/prot_mw.html).

### 2.7 Quantitative PCR

Total RNA was extracted from patient‐derived BCLs using a *mir*Vana miRNA Isolation Kit (Invitrogen, #AM1561) and DNase‐treated using a Turbo DNA‐*free* Kit (Invitrogen, #AM1907). BCL RNA was reverse transcribed into cDNA using a SuperScript VILO cDNA Synthesis Kit (Invitrogen, #11754050). *RAD51C* gene expression was assessed in BCL cDNA using two TaqMan Gene Expression Assays (Applied Biosystems) targeting *RAD51C* exons 3 and 4 (Hs00980052_m1) and exons 5 and 6 (Hs00980054_m1). Quantitative PCR was conducted using TaqMan Fast Advanced Master Mix (Applied Biosystem, #4444556) on a ViiA 7 Real‐Time PCR System (Applied Biosystems). *ACTB* (Hs01060665_g1) was used as an internal control. Six samples from three independent experiments were run in duplicate, and results were analyzed using the comparative threshold cycle (∆∆_CT_) method.

### 2.8 Immunoblotting

Whole cell lysates were prepared in RIPA buffer from BCL as previously described (Mostafa et al., 2014). Proteins were quantified using a Pierce BCA Protein Assay Kit (Thermo Scientific, #23225) and reduced with 2‐mercaptoethanol (Gibco, #21985023). RAD51C protein expression was assessed in BCL whole cell lysates by electrophoresing 25 µg of protein by 10% SDS‐PAGE, followed by western blotting. Membranes were blocked in 5% milk powder in TBS‐Tween (0.15 M NaCl, 0.05 M Tris pH 7.4, 0.05% Tween 20) for 1 hr and incubated overnight with 4 µg/ml monoclonal mouse anti‐RAD51C (2H11) antibody (Santa Cruz Biotechnology, #sc‐56214) or 250 ng/ml mouse anti‐alpha tubulin (B‐7) antibody (Santa Cruz Biotechnology, #sc‐5286) at 4°C. Antibody binding was detected with 1:10,000 dilutions of horseradish peroxidase‐conjugated goat anti‐mouse secondary antibody (Jackson Immunoresearch, #115‐036‐071) and Clarity Western ECL substrate (Bio‐Rad, #1705060). Immunoreactivity was visualized and quantified by scanning densitometry using an ImageQuant LAS 4000 and ImageQuant TL Software v8.1, respectively (GE Healthcare). Five independent experiments were performed, and RAD51C protein expression was normalized to that of alpha tubulin.

### 2.9 Loss‐of‐heterozygosity analysis

Tumor DNA was obtained from available formalin‐fixed paraffin‐embedded (FFPE) blocks of breast (family 5440; PID: III‐6) and ovarian (family 5088; PID: II‐1) tumors and sequenced for *RAD51C* c.571 + 4A > G as previously described. Tumor tissue sections were stained with H&E and cell type, primary site, grade and clinical staging was confirmed by a pathologist (RN). Genomic DNA was isolated from 10 µm unstained serial tissue sections of FFPE tumor blocks using a QIAamp DNA FFPE Tissue Kit (Qiagen, #56404). The percentage of tumor cells versus nontumor cells (including inflammatory cells, fibroblasts, and macrophages) was determined for each case by manually counting cells from 10 representative areas at 20× magnification.

### 2.10 Statistical analysis

Statistical analyses of mRNA and protein expression were performed using Prism v7.00 (GraphPad Software) with *p* < .05 considered statistically significant.

## 3 RESULTS

### 3.1 Recurrent *RAD51C* c.571 + 4A > G variant co‐segregates with both BC and OC

The index case (family 5440; PID: III‐6) was diagnosed with BC (invasive carcinoma of no special type (invasive ductal carcinoma, not otherwise specified), clinical stage IIA, estrogen receptor (ER) negative, progesterone receptor (PR) negative, and human epidermal growth factor receptor 2 (HER2) negative) at age 49, and screened negative for pathogenic and likely pathogenic variants in moderate‐ and high‐risk cancer susceptibility genes. Whole‐exome sequencing identified 66 variants that passed filtering criteria (excluding *RAD51C* c.571 + 4A > G) with 16 variants associated with cancer, specifically with tumor progression only and were excluded from further investigation (Table S4). Of the 40 copy number variants identified in the index case, all were classified as either benign or likely benign (data not shown). At this point, the VUS identified in the *RAD51C* gene (NM_058216.3: *RAD51C* c.571 + 4A > G; rs587780257) represented the most promising candidate genetic variant to investigate further. Cascade screening on maternal and paternal sides demonstrated that, despite the propensity toward maternal cancers, the *RAD51C* c.571 + 4A > G variant was inherited from the proband's father (PID: II‐6) (Figure 1a). Her deceased paternal uncle (PID II‐5) was diagnosed with pancreatic cancer at age 68 and her paternal grandfather had a history of prostate cancer. The proband's mother (PID: II‐7) and other available maternal relatives are wild‐type; therefore, we concluded that the colon, leukemia and other cancers are not attributed to the *RAD51C* c.571 + 4A > G variant. In summary, the *RAD51C*‐associated cancer in family 5440 is BC under age 50. The *RAD51C* variant has been transmitted through the paternal side of the family with an apparent autosomal dominant mode of inheritance. The proband's father (II‐6) is a heterozygote and cancer‐free at age 78.

The deceased proband (PID: II‐1) in family 5088 (Figure 1b) was diagnosed with OC (clinical stage IV HGSC) at age 56. The proband's father (I‐1) is alive at age 94 and there is no information on the paternal side of this family. However, there is strong family history of relevant cancer on the maternal side. The proband's deceased mother (PID I‐2) was diagnosed with OC at age 54, and the proband's deceased maternal aunt (PID I‐5) was diagnosed with brain cancer at age 50. Three of the proband's sisters are also *RAD51C* c.571 + 4A > G heterozygotes, including one diagnosed with BC (invasive lobular carcinoma, clinical stage II) diagnosed at age 48 (PID: II‐3). One daughter of PID II‐3 was diagnosed with colon cancer at age 33. The two unaffected sisters (PID: II‐5; and PID: II‐7) are cancer free at ages 72 and 62 respectively. However, the 72‐year‐old sister had a bilateral salpingo‐oophorectomy (BSO) at age 37 for benign indications. She transmitted *RAD51C* c.571 + 4A > G to her daughter diagnosed with BC (invasive ductal carcinoma, clinical stage I, ER, PR, and Her2 positive) at age 42 (PID: III‐4). The proband's brother (PID: II‐2) inherited *RAD51C* c.571 + 4A > G and is cancer free at age 76. In summary, the *RAD51C*‐associated cancer in family 5088 is BC under age 50. The *RAD51C* variant is likely transmitted through the maternal side in an autosomal dominant pattern. There is a male (II‐3) and a female (II‐7) who carry *RAD51C* c.571 + 4A > G and are cancer‐free at ages 76 and 62 respectively.

Family 05011 is a large family with numerous cancers in addition to BC and OC (Figure 1c). The proband (PID: II‐18) recently diagnosed with BC (invasive ductal carcinoma, clinical stage IA ER, PR, Her2 positive), was previously diagnosed with endometrial cancer (clinical stage IB) at age 75 and colon cancer at age 70. The proband's mother (PID I‐7) was diagnosed with BC at age 49. The proband's niece (PID: III‐23) was diagnosed with BC (invasive ductal carcinoma, clinical stage II, ER, PR positive) at age 53, and the proband's brother was unavailable as he died at age 26 but was previously diagnosed with testicular cancer. Although not all samples were available for genotyping, family history shows two first cousins on the proband's maternal side, one diagnosed with HGSC at age 48 (PID: II‐20) and another diagnosed with HGSC (clinical stage IIIC) at age 67 (PID: II‐22) and is a heterozygote, transmitting *RAD51C* c.571 + 4A > G to her daughter (PID: III‐33) who was diagnosed with BC (invasive ductal carcinoma, clinical stage III, ER, PR, and HER2 negative) at age 37 and HGSC (clinical stage IIIB) at age 41. In summary, the *RAD51C*‐associated cancer in family 05011 is BC (age range: 37–77) and OC (age range: 41–67). The *RAD51C* variant is transmitted through the maternal side with an apparent autosomal dominant mode of inheritance. Three female heterozygotes are cancer‐free at ages 49 (PID: III‐27), 65 (PID: III‐26), and 72 (PID: II‐21) with another female (PID: II‐19) diagnosed with pancreatic cancer at age 83. Several relatives (PID: III‐15; III‐24; III‐28) with other types of cancers (endometrioid, thyroid, kidney) screened negative for the *RAD51C* c.571 + 4A > G variant.

The proband (PID: III‐4) in family 22427 (Figure 1c), was diagnosed with HGSC (clinical stage III) at age 67. Her sister (PID III‐3) was diagnosed with OC (unknown subtype) at age 47, her son (PID IV‐2) was diagnosed with throat/tongue cancer at age 48, and her cousin (PID IV‐1) was diagnosed with OC (age/subtype unknown). In summary, the *RAD51C*‐associated cancer in family 22427 is OC (age 67). It is unclear from which side of the family the proband inherited the *RAD51C* c.571 + 4A > G variant. One female heterozygote is cancer‐free at age 68 (PID: III‐5).

The proband (PID: III‐4) in family 7214 (Figure 1d) was diagnosed with HGSC (clinical stage III) at age 66. Although unavailable for genotyping, her paternal grandmother (I‐2) was diagnosed with OC (age/subtype unknown) and died at age 57, and her deceased father (II‐6) was diagnosed with colorectal cancer. There is no evidence of HBOC on the maternal side. The proband transmitted the variant to her daughter (PID: IV‐2) who is cancer‐free at age 48. In summary, the *RAD51C*‐associated cancer in family 7214 is OC (age 66). The *RAD51C* variant is likely transmitted through the paternal side of the family in an autosomal dominant pattern.

In summary, the recurrent *RAD51C* c.571 + 4A > G variant is associated with cancers that occur in two or more heterozygotes include BC, diagnosed in five females (age range: 37–77) and OC, diagnosed in four females (age range: 41–67). In addition, a single female was diagnosed with both BC (37 years old) and OC (41 years old) (Table 1; Figure 1). In *RAD51C* c.571 + 4A > G heterozygotes, mean age of diagnosis of BC or OC occurred at age 51 and 59.4 respectively. Invasive ductal breast carcinoma and HGSC represent the most common histotypes for *RAD51C*‐associated breast and ovarian cancer respectively. The inheritance pattern of the *RAD51C* variant is consistent with autosomal dominant with reduced penetrance. Although three males (including one obligate carrier) were identified, no cases of male breast cancer were observed. Additionally, seven *RAD51C* heterozygotes (two males; five females) are cancer‐free (age range: 48–78).

**Table 1:** Clinical characteristics of *RAD51C* c.571 + 4A > G heterozygotes with a cancer diagnosis

Family/individual	Tumor type	Age of onset	Tumor histology	Stage	Receptor Status	Treatment
05011						
II‐18	Colon	70	–	–	–	Chemotherapy
	Endometrial	75	Endometrioid	IB	–	Surgery
	Breast	77	Ductal	IA	ER + PR+Her2+	Lumpectomy, chemotherapy
II‐19	Pancreas	83	–	IV	–	Chemotherapy
II‐22	Ovary	67	HGS	IIIC	–	Neo‐adj. chemotherapy, debulking
III‐23	Breast	53	Ductal	II	ER + PR+	Mastectomy, radiation
III‐33	Breast	37	Ductal	III	Triple negative	Neo‐adj. chemotherapy, lumpectomy
	Ovary	41	HGS	IIIB	–	Debulking, chemotherapy
5440						
III‐6	Breast	49	Ductal	IIA	Triple negative	Mastectomy, chemotherapy
5088						
II‐1	Ovary	56	HGS	IV	–	Debulking, chemotherapy
II‐3	Breast	48	Lobular	II	–	Mastectomy, chemotherapy
III‐4	Breast	42	Ductal	I	ER + PR+Her2+	Mastectomy
7214						
III‐4	Ovary	66	HGS	III	–	Neo‐adj. chemotherapy, debulking
22427						
III‐4	Ovary	67	HGS	III	–	Neo‐adj. chemotherapy, debulking

### 3.2 Haplotype, case‐control and population analysis

Haplotype analysis flanking 5.29 Mbp on 17q at the *RAD51C* locus was performed in all samples testing positive for the *RAD51C* c.571 + 4A > G variant. Comparison of HBOC‐associated haplotypes across families revealed a shared, extended 5.07 Mbp region (Table 2). Genotype analysis revealed a carrier frequency of 0.331% (3/907 individuals), and an estimated minor allele frequency (MAF) of 0.165% (3/1,814 alleles) in the NL population compared with 0.0032% (1/31,412) in the general Caucasian population (Genome Aggregation Database (Lek et al., 2016)), a 52‐fold increase in this genetic isolate. Interestingly, the HBOC‐associated alleles were also identified in an incident BC case and three population controls carrying the *RAD51C* c.571 + 4A > G variant, suggesting a common ancestor. Review of genealogical and census records have revealed that the first known individuals linked via pedigree study in this investigation immigrated to outport Newfoundland from southwest England around 1,750. Despite extensive pedigree review using all available resources, we were unable to link the five multiplex families in this investigation beyond the association of families 05011 and 22427 by marriage, and so it is likely that the variant arose in a common ancestor prior to available records shortly before Newfoundland's colonization. The estimated mutation age was calculated using the linkage disequilibrium and recombination fraction between *RAD51C* and markers D17S1606 and D17S808. Assuming an average generation age of 25 years (Milting et al., 2015), this variant is estimated to have arose between 397 and 1692 years ago, corresponding to between the years 257 and 1552 based on the average proband year of birth of 1949. We interpret these results as evidence of a strong founder effect for *RAD51C* c.571 + 4A > G.

**Table 2:** Haplotypes of *RAD51C* c.571 + 4A > G heterozygotes with the minimum disease‐associated haplotype (5.07 Mbp) indicated in bold

Marker	5440		5088					22427	05011							7214		Population Controls			BC Case
	III‐6^a^	II‐7	II‐2	II‐3	II‐7	II‐1^a^	II‐5	III‐4^a^	II‐18^a^	III‐23	II‐19	II‐22	III‐27	III‐26	II‐21	IV‐2	III‐4^a^	2‐0198	NF‐C‐15	11795	093
*D17S666*	161	161	161	161	161	161	161	157	157	157	165	165	165	165	165	165	165	(165)	(165)	(165)	(165)
*D17S1853*	142	142	142	142	142	142	142	142	142	142	142	142	142	142	142	142	142	(142)	(142)	(142)	(142)
*D17S1606*	164	164	164	164	164	164	164	128	128	128	164	164	164	164	164	164	164	(164)	(164)	(164)	(164)
***D17S1161***	**173**	**173**	**173**	**173**	**173**	**173**	**173**	**173**	**173**	**173**	**173**	**173**	**173**	**173**	**173**	**173**	**173**	(**173**)	(**173**)	(**173**)	(**173**)
***RAD51C* c.571 + 4**	**G**	**G**	**G**	**G**	**G**	**G**	**G**	**G**	**G**	**G**	**G**	**G**	**G**	**G**	**G**	**G**	**G**	**G**	**G**	**G**	**G**
***D17S1604***	**94**	**94**	**94**	**94**	**94**	**94**	**94**	**94**	**94**	**94**	**94**	**94**	**94**	**94**	**94**	**94**	**94**	(**94**)	(**94**)	(**94**)	(**94**)
***D17S923***	**289**	**289**	**289**	**289**	**289**	**289**	**289**	**289**	**289**	**289**	**289**	**289**	**289**	**289**	**289**	**289**	**289**	(**289**)	(**289**)	(**289**)	(**289**)
*D17S808*	142	142	142	142	142	142	142	142	142	142	154	154	154	154	154	142	142	(142)	(142)	(142)	(142)
*D17S794*	224	224	224	224	224	224	224	224	224	224	230	230	230	230	230	224	224	(224)	(224)	(224)	(224

a Denotes the proband within each family. The disease‐associated haplotype for the population controls and breast cancer (BC) case are inferred.

### 3.3 Tumour profiling of *RAD51C* c.571 + 4A > G heterozygotes

**Figure 2:**
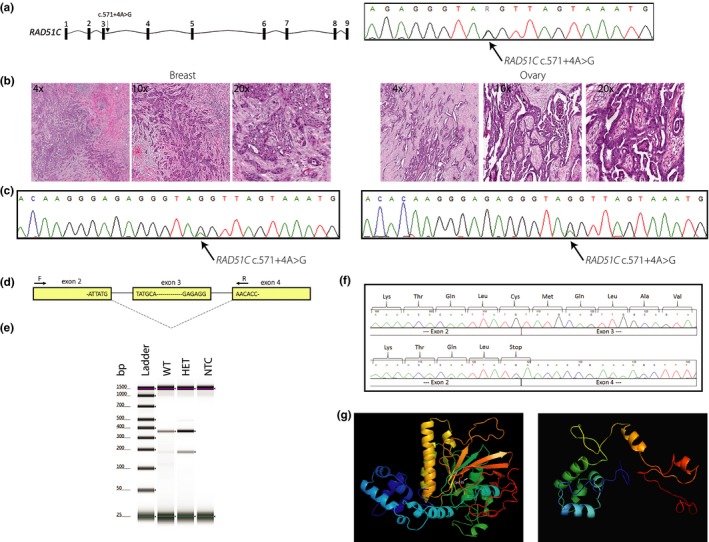
Germline and somatic analyses of the *RAD51C* c.571 + 4A > G splicing variant causing exon skipping. (a) Schematic illustrating the nine exons comprising the *RAD51C* gene with the location (left) and representative Sanger sequence of the c.571 + 4A > G variant indicated with an arrow (right). (b) H&E staining of breast tumor sections from an affected proband (family 5440; PID: III‐6; left) and ovary tumor sections from an affected proband (family 5088; PID: II‐1; right) at 4x, 10x and 20x magnification. (c) A representative Sanger sequence of genomic DNA of breast tumor (left) and ovary tumor (right) depicting the c.571 + 4A > G variant homozygosity indicated with an arrow [Correction added on 14 January 2020, after first online publication: the preceding two sentences have been updated to reflect the correct legends for Figures 2b and 2c.]. The presence of a minor wild‐type allele is attributed to the presence of inflammatory cells in the tumor specimens. (d) Schematic illustrating alternative splicing due to the *RAD51C* c.571 + 4A > G variant and the location of primers used for the cDNA analysis. (e) TapeStation gel image of RT‐PCR analysis illustrating three bands in a *RAD51C* c.571 + 4A > G heterozygote (family 5440; PID: III‐6) compared with a single band in a related wild‐type individual (family 5440; PID: II‐7) using BCL samples. (f) Sanger sequencing of cDNA in a *RAD51C* c.571 + 4A > G heterozygote (family 5440; PID: III‐6) compared with a related wild‐type individual (family 5440; PID: II‐7) illustrating the skipping of exon 3. (g) In silico modeling using Phyre2 depicts the structure of the normal RAD51C protein (left), with the mutated residue (p.Cys135) labeled, and the predicted effect of the *RAD51C* c.571 + 4A > G variant (right), which creates a severely truncated protein lacking critical domains required for HR repair. The Phyre2 software was able to model 86% and 95% of the wild‐type and mutant RAD51C protein structures at >90% confidence, respectively; model confidence is indicated by color from high (red) to low (blue) [Correction added on 14 January 2020, after first online publication: Figure 2 has been updated to show the correct histology images.]

To investigate loss‐of‐heterozygosity, tissues from a metastatic HGSC (family 5088; PID: II‐1) and a BC (family 5440; PID: III‐6) were analyzed (Figure 2b). Pathology review revealed a well‐ to moderately differentiated papillary serous ovarian carcinoma and an invasive ductal breast carcinoma of no special type. The HGSC specimen had a tumor cell percentage of 48.4% (2,736/5,650) whereas the invasive ductal breast carcinoma had a tumor cell percentage of 86.3% (5010/5804). The peak intensity of the *RAD51C* c.571 + 4A > G variant was considerably reduced in both specimens compared with DNA derived from blood and represents nontumor cells (inflammatory cells, fibroblasts, and macrophages) in both specimens (Figure 2b and c). On average, 70.5% and 68.3% of peak signal intensity at the c.571 + 4 position was attributed to the disease‐associated allele in the HGSC and BC specimens respectively.

### 3.4 *RAD51C* c.541 + 4A > G: RNA and protein analysis

**Figure 3:**
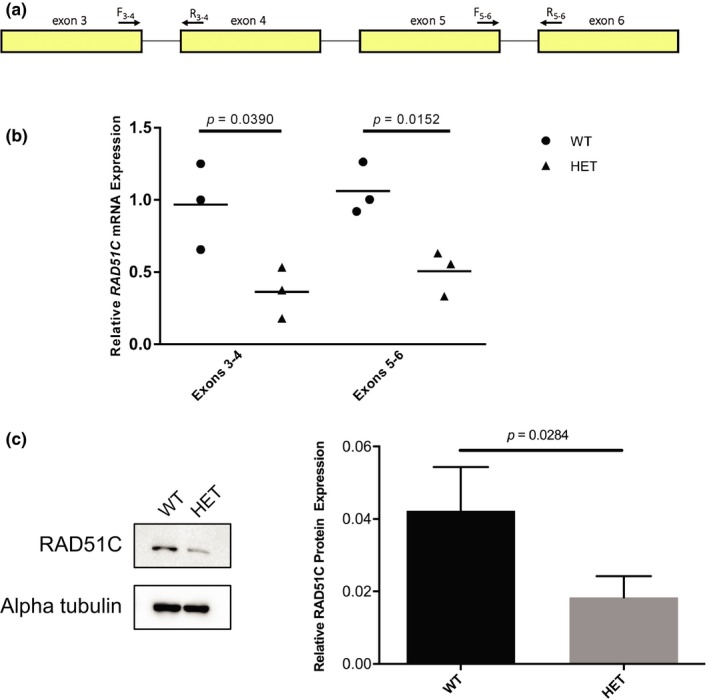
Gene and protein expression analysis of *RAD51C*. (a) Schematic illustrating the location of primers used for the gene expression analysis. (b) The relative expression of *RAD51C* mRNA, determined by quantitative PCR analysis of patient‐derived BCL samples, revealed significantly lower *RAD51C* mRNA expression from an affected proband (family 5440; PID: III‐6; HET) compared with a related wild‐type individual (family 5440; PID: II‐7; WT), regardless of which exons were assayed (exons 3–4, *p* = .0390; exons 5–6, *p* = .0152). *ACTB* was used as an internal control. Data shown are representative of three independent experiments run in duplicate and averaged. *p* values were calculated by unpaired *t*‐test. (c) RAD51C protein expression in RIPA lysates from patient‐derived BCL samples including representative western blot (left) and densitometric analysis results (right) revealed significantly lower RAD51C protein expression from an affected proband (family 5440; PID: III‐6; HET) compared with a related wild‐type individual (family 5440; PID: II‐7; WT; *p* = .0284). Alpha tubulin was used as a loading control and probed for on the same blot as RAD51C. Data shown are representative of five independent experiments. *p* values were calculated by paired *t*‐test. Mean ± *SEM* is presented.

*RAD51C* has 17 annotated transcripts in GTEx (GTEx Consortium, 2013), one extra transcript annotated in Ensembl, and two validated RefSeq transcripts, with NM_058216.3 being the most commonly expressed transcript in EBV‐transformed lymphocytes as well as breast and ovary tissue (GTEx Consortium, 2013). In silico splicing analyses predicted that *RAD51C* c.541 + 4A > G, which resides in the 5′ of intron 3 (NM_058216.3), decreases the affinity for the canonical donor splice site resulting in exon skipping (Table S5). The RNA and protein analyses were performed using BCL from a heterozygote (family 5440; PID: III‐6) and a related wild‐type individual (family 5440; PID: II‐7). PCR analysis of patient‐derived cDNA using primers specific to *RAD51C* exons 2 and 4 (Figure 2d) demonstrated loss of *RAD51C* exon 3 in the heterozygote compared with the wild‐type individual (Figure 2e). TOPO TA cloning of cDNA amplicons showed a considerable reduction in transcripts containing exon 3 in the heterozygote (data not shown). Complete loss of the 167 bp exon 3 is predicted to cause an immediate premature termination codon (NP_478123.1: p.Cys135Ter; Figure 2f) and a truncated protein lacking all residues after amino acid 134 including the Walker B motif (amino acids 238–242) and the nuclear localization signal (amino acids 366–370) (Figure 2g). Next, we quantified gene and protein expression. As *RAD51C* c.541 + 4A > G resides in intron 3, we designed primers targeting exons 3 and 4 (Figure 3a), revealing a significant decrease (62.9%) in *RAD51C* expression in the heterozygote (*p* = .0390; Figure 3b). We hypothesized that loss of exon 3 and creation of an immediate premature stop codon would trigger nonsense‐mediated mRNA decay. Therefore, we next targeted exons 5 and 6, which were also significantly decreased (51.9%) in the heterozygote (*p* = .0152; Figure 3b). At the protein level, western blot analysis showed a significant decrease (56.5%) in the heterozygote (*p* = .0284; Figure 3c). Furthermore, no bands approximating the 14.9 kDA RAD51C isoform 2 (NP_002867) were detected experimentally using a polyclonal anti‐RAD51C antibody (Figure S1).

Findings from this study provide additional information to re‐classify *RAD51C* c.571 + 4A > G as a pathogenic variant (PS3, PS4, PM2, PP1, PP3), according to the ACMG guidelines (Richards et al., 2015).

## 4 DISCUSSION

We have determined that a recurrent *RAD51C* splicing variant (c.571 + 4A > G) predisposes to both BC and OC in the NL population due to strong founder effect. Furthermore, *RAD51C* c.571 + 4A > G is increased ~52‐fold in the NL population and all positive heterozygotes in the population share an extended HBOC‐associated haplotype, suggesting a strong founder effect. The *RAD51C* c.571 + 4A > G phenotype includes both BC and OC. For example, we observed five female heterozygotes with BC only and four with OC only. There was also one female heterozygote diagnosed with both BC and OC, suggesting that this pathogenic variant increases the risk of BC independent of OC. Functional studies using patient‐derived cells demonstrate that *RAD51C* c.571 + 4A > G alters gene splicing and reduces gene and protein expression. Sequencing of ovary and breast tumor samples from two *RAD51C* c.571 + 4G > A probands indicates loss‐of‐heterozygosity and suggests loss of function in the affected tissue represents a “second hit”, setting the stage for oncogenesis.

To estimate penetrance with confidence in family‐based studies, it is important to strive for complete ascertainment to circumvent errors due to ascertainment biases and low sample number. Therefore, we were not able to estimate penetrance, as more time and effort is required to recruit across these five extended pedigrees. Other studies investigating the association of *RAD51C* pathogenic variants in HBOC cases provide strong evidence supporting germline deleterious variants in *RAD51C* conferring predisposition to OC with a cumulative risk of 9% by age 80 (Blanco et al., 2014; Clague et al., 2011; Eoh et al., 2018; Jonson et al., 2016; Loveday et al., 2012; Lu et al., 2018; Meindl et al., 2010; Neidhardt et al., 2017; Osorio et al., 2012; Pang et al., 2011; Pelttari et al., 2011, 2018; Romero et al., 2011; Sanchez‐Bermudez et al., 2018; Schnurbein et al., 2013; Song et al., 2015; Sung et al., 2017; Thompson et al., 2012; Vuorela et al., 2011). In our study, we observed more BC only patients compared with OC only patients in heterozygotes, which is consistent with other studies of BC only families (Blanco et al., 2014; Jonson et al., 2016; Meindl et al., 2010; Neidhardt et al., 2017; Osorio et al., 2012; Pelttari et al., 2018; Sanchez‐Bermudez et al., 2018; Schnurbein et al., 2013; Yablonski‐Peretz et al., 2016). In an unbiased French study of 2,063 consecutive patients diagnosed with HBOC, three *RAD51C* pathogenic variants were identified in BC only cases (Golmard et al., 2017). However, in a larger unbiased study of 65,057 BC only patients using multigene sequencing, *RAD51C* pathogenic variants were not associated with an increased risk (Couch et al., 2017). In summary, there is a well‐established increased risk of *RAD51C* pathogenic variants for OC; however, whether or not there is an increased risk for BC only remains uncertain.

In this study, BC occurs at a mean age of diagnosis of 49.2 and ovarian cancer occurs at a mean age of diagnosis of 59.4 in *RAD51C* c.571 + 4G > A heterozygotes, and is consistent with a review of 19 studies investigating *RAD51C* variants in HBOC families, which revealed that *RAD51C*‐associated BC (*n* = 56) occurs with a mean age of diagnosis of 48.9 years (range: 27–76) whereas *RAD51C*‐associated OC (*n* = 31) occurs with a mean age of diagnosis of 59.1 years (range: 42–81). *RAD51C* c.571 + 4A > G heterozygotes diagnosed with OC all exhibited HGSC, and heterozygotes diagnosed with BC primarily exhibited invasive ductal carcinoma. Individuals with pathogenic *RAD51C* variants in BC were previously reported to be primarily ductal, hormone receptor‐positive and HER2‐negative, or triple negative invasive carcinomas of no special type, diagnosed at an early stage with moderate differentiation (Gevensleben et al., 2014; Meindl et al., 2010; Schnurbein et al., 2013), and OC is primarily HGSC (Cunningham et al., 2014; Gevensleben et al., 2014). In addition, we report occurrence of pathologic *RAD51C* variants in triple positive BC which has not been previously reported. Compared with *BRCA1* phenotype, *RAD51C*‐associated OC occurs at an older mean age of diagnosis (59.4 vs. 54) but at a similar age as *BRCA2* (59.4 vs. 59.5) (Kuchenbaecker et al., 2017). *RAD51C*‐associated BC in this study occurs at an older mean age of diagnosis compared with *BRCA1* (49.2 vs. 44) or *BRCA2* (49.2 vs. 48) (Kuchenbaecker et al., 2017). It is evident that *RAD51C* pathogenic variants may be associated with other cancers as previously reviewed (Sopik et al., 2015). In this study, although other cancers were noted in the extended pedigrees, the extent to which the *RAD51C* c.571 + 4A > G variant underlies these various cancers requires further clinical recruitment and genotyping. Determining the phenotypic spectrum of *RAD51C*, including the recurrent variant identified in this population, requires a larger number of heterozygotes.

Given that the association of *RAD51C* with HBOC is relatively new and the frequency of pathogenic variants relatively low, using the NL genetic isolate is a powerful method to identify how the recurrent *RAD51C* c.571 + 4A > G variant is transmitted across families and through time. Based on family studies and functional analysis, we provide sufficient evidence that the recurrent variant, currently reported as a VUS, should be clinically reclassified as pathogenic according to ACMG guidelines. Furthermore, the NL population frequency of this variant is increased ~52‐fold compared with the general Caucasian population, and disease allele sharing with population controls, provides evidence of a recent common ancestor and a strong founder effect. Estimates of the variant age suggest it arose in a common ancestor in the English population prior to NL settlement. Reports of pathogenic founder variants in *RAD51C* have also been noted in Finnish (c.93delG and c.837 + 1G > A) and Swedish (c.774delT) populations (Osorio et al., 2012; Pelttari et al., 2012; Romero et al., 2011; Vuorela et al., 2011).

Pathogenic splicing variants in *RAD51C* are commonly associated with HBOC. For example, *RAD51C* c.837 + 1G > A results in exon 5 skipping (Pelttari et al., 2011) with a different variant resulting in exon 6 skipping (Meindl et al., 2010), and a novel pathogenic splice‐site *RAD51C* variant at the last nucleotide of exon 2 (c.404G > C/T) in BC and OC patients in families (Neidhardt et al., 2017). Currently, *RAD51C* c.541 + 4A > G is clinically classified as a VUS, even though it was previously reported (Shirts et al., 2016) in a BC patient diagnosed at age 60 (supplementary text) and results in exon 3 skipping. Within the 5′ splice donor site of intron 3, Pang *et al*. identified a substitution in the adjacent base (c.571 + 5G > A) in females with familial BC (Pang et al., 2011), and a newborn diagnosed with Fanconi anemia had c.571 + 5G > A *in trans* with c.935G > A (Jacquinet et al., 2018). Experimental evidence in patient‐derived samples confirmed in silico predictions that *RAD51C* c.541 + 4A > G disrupts slicing, specifically skipping exon 3 of *RAD51C*, creating an immediate premature stop codon and triggering nonsense mediated decay.

The population‐based approach used in this study to investigate the recurrent *RAD51C* c.571 + 4A > G VUS confirm that: (a) *RAD51C* c.571 + 4A > G is pathogenic; (b) *RAD51C* c.571 + 4A > G is over represented in NL HBOC patients; (c) *RAD51C* is associated with BC in addition to OC in these families; and (d) *RAD51C* should be considered for inclusion on multigene panels for incident BC and OC cases in NL. Although pathogenic variants have been identified in individuals with other cancer types, the phenotypic spectrum of *RAD51C* in cancer remains to be fully determined. Extrapolation of the MAF suggests there are 1,500 at‐risk *RAD51C* c.571 + 4A > G heterozygotes in NL (population size = 519,000). This represents a considerable number of at‐risk individuals for an otherwise extremely rare variant globally. The *RAD51C* c.571 + 4A > G variant may be relevant to individuals of NL ancestry and may not be a major HBOC predisposition pathogenic variant in other populations; however, it is clear that *RAD51C* c.571 + 4A > G, and perhaps other yet undiscovered founder variants, may explain the increased incidence and perhaps mortality associated with HBOC in NL.

## CONFLICT OF INTEREST

The authors declare no potential conflicts of interest.

## References

[mgg31070-bib-0001] Abdelfatah, N. , McComiskey, D. A. , Doucette, L. , Griffin, A. , Moore, S. J. , Negrijn, C. , … Young, T.‐L. (2013). Identification of a novel in‐frame deletion in KCNQ4 (DFNA2A) and evidence of multiple phenocopies of unknown origin in a family with ADSNHL. European Journal of Human Genetics, 21(10), 1112–1119. 10.1038/ejhg.2013.5 23443030 PMC3778362

[mgg31070-bib-0002] Blanco, A. , Gutiérrez‐Enríquez, S. , Santamariña, M. , Montalban, G. , Bonache, S. , Balmaña, J. , … Vega, A. (2014). RAD51C germline mutations found in Spanish site‐specific breast cancer and breast‐ovarian cancer families. Breast Cancer Research and Treatment, 147(1), 133–143. 10.1007/s10549-014-3078-4 25086635

[mgg31070-bib-0003] Canadian Cancer Statistics (2017). Canadian Cancer Society’s Advisory Committee on Cancer Statistics. Available at: http://cancer.ca/Canadian-Cancer-Statistics-2017-EN.pdf.

[mgg31070-bib-0004] Capoluongo, E. , Ellison, G. , López‐Guerrero, J. A. , Penault‐Llorca, F. , Ligtenberg, M. J. L. , Banerjee, S. , … de Castro, D. G. (2017). Guidance statement on BRCA1/2 tumor testing in ovarian cancer patients. Seminars in Oncology, 44(3), 187–197. 10.1053/j.seminoncol.2017.08.004 29248130

[mgg31070-bib-0005] Chun, J. , Buechelmaier, E. S. , & Powell, S. N. (2013). Rad51 paralog complexes BCDX2 and CX3 act at different stages in the BRCA1‐BRCA2‐dependent homologous recombination pathway. Molecular and Cellular Biology, 33(2), 387–395. 10.1128/MCB.00465-12 23149936 PMC3554112

[mgg31070-bib-0006] Clague, J. , Wilhoite, G. , Adamson, A. , Bailis, A. , Weitzel, J. N. , & Neuhausen, S. L. (2011). RAD51C germline mutations in breast and ovarian cancer cases from high‐risk families. PLoS ONE, 6(9), e25632 10.1371/journal.pone.0025632 21980511 PMC3182241

[mgg31070-bib-0007] Couch, F. J. , Shimelis, H. , Hu, C. , Hart, S. N. , Polley, E. C. , Na, J. , … Dolinsky, J. S. (2017). Associations between cancer predisposition testing panel genes and breast cancer. JAMA Oncology, 3(9), 1190–1196. 10.1001/jamaoncol.2017.0424 28418444 PMC5599323

[mgg31070-bib-0008] Cunningham, J. M. , Cicek, M. S. , Larson, N. B. , Davila, J. , Wang, C. , Larson, M. C. , … Goode, E. L. (2014). Clinical characteristics of ovarian cancer classified by BRCA1, BRCA2, and RAD51C status. Scientific Reports, 4, 4026 10.1038/srep04026 24504028 PMC4168524

[mgg31070-bib-0009] Eoh, K. J. , Kim, J. E. , Park, H. S. , Lee, S.‐T. , Park, J. S. , Han, J. W. , … Nam, E. J. (2018). Detection of germline mutations in patients with epithelial ovarian cancer using multi‐gene panels: Beyond BRCA1/2. Cancer Research and Treatment, 50(3), 917–925. 10.4143/crt.2017.220 29020732 PMC6056949

[mgg31070-bib-0010] Gevensleben, H. , Bossung, V. , Meindl, A. , Wappenschmidt, B. , de Gregorio, N. , Osorio, A. , … Schmutzler, R. K. (2014). Pathological features of breast and ovarian cancers in RAD51C germline mutation carriers. Virchows Archiv, 465(3), 365–369. 10.1007/s00428-014-1619-1 24993905

[mgg31070-bib-0011] Golmard, L. , Castéra, L. , Krieger, S. , Moncoutier, V. , Abidallah, K. , Tenreiro, H. , … Houdayer, C. (2017). Contribution of germline deleterious variants in the RAD51 paralogs to breast and ovarian cancers. European Journal of Human Genetics, 25(12), 1345–1353. 10.1038/s41431-017-0021-2 29255180 PMC5865182

[mgg31070-bib-0012] Green, R. C. , Narod, S. A. , Morasse, J. , Young, T. L. , Cox, J. , Fitzgerald, G. W. , … Poitras, P. . (1994). Hereditary nonpolyposis colon cancer: Analysis of linkage to 2p15‐16 places the COCA1 locus telomeric to D2S123 and reveals genetic heterogeneity in seven Canadian families. American Journal of Human Genetics, 54(6), 1067–1077. 8198129 PMC1918192

[mgg31070-bib-0013] GTEx Consortium (2013). The Genotype‐Tissue Expression (GTEx) project. Nature Genetics, 45(6), 580–585. 10.1038/ng.2653 23715323 PMC4010069

[mgg31070-bib-0014] Halldorsson, B. V. , Palsson, G. , Stefansson, O. A. , Jonsson, H. , Hardarson, M. T. , Eggertsson, H. P. , … Stefansson, K. (2019). Characterizing mutagenic effects of recombination through a sequence‐level genetic map. Science, 363(6425), 10.1126/science.aau1043 30679340

[mgg31070-bib-0015] Jacquinet, A. , Brown, L. , Sawkins, J. , Liu, P. , Pugash, D. , Van Allen, M. I. , & Patel, M. S. (2018). Expanding the FANCO/RAD51C associated phenotype: Cleft lip and palate and lobar holoprosencephaly, two rare findings in Fanconi anemia. European Journal of Medical Genetics, 61(5), 257–261. 10.1016/j.ejmg.2017.12.011 29278735

[mgg31070-bib-0016] Jønson, L. , Ahlborn, L. B. , Steffensen, A. Y. , Djursby, M. , Ejlertsen, B. , Timshel, S. , … Hansen, T. V. O. (2016). Identification of six pathogenic RAD51C mutations via mutational screening of 1228 Danish individuals with increased risk of hereditary breast and/or ovarian cancer. Breast Cancer Research and Treatment, 155(2), 215–222. 10.1007/s10549-015-3674-y 26740214

[mgg31070-bib-0017] Kelley, L. A. , Mezulis, S. , Yates, C. M. , Wass, M. N. , & Sternberg, M. J. (2015). The Phyre2 web portal for protein modeling, prediction and analysis. Nature Protocols, 10(6), 845–858. 10.1038/nprot.2015.053 25950237 PMC5298202

[mgg31070-bib-0018] Kuchenbaecker, K. B. , Hopper, J. L. , Barnes, D. R. , Phillips, K. A. , Mooij, T. M. , Roos‐Blom, M. J. , … Olsson, H. (2017). Risks of breast, ovarian, and contralateral breast cancer for BRCA1 and BRCA2 mutation carriers. JAMA, 317(23), 2402–2416. 10.1001/jama.2017.7112 28632866

[mgg31070-bib-0019] Lek, M. , Karczewski, K. J. , Minikel, E. V. , Samocha, K. E. , Banks, E. , Fennell, T. , … MacArthur, D. G. (2016). Analysis of protein‐coding genetic variation in 60,706 humans. Nature, 536(7616), 285–291. 10.1038/nature19057 27535533 PMC5018207

[mgg31070-bib-0020] Lord, C. J. , & Ashworth, A. (2016). BRCAness revisited. Nature Reviews Cancer, 16(2), 110–120. 10.1038/nrc.2015.21 26775620

[mgg31070-bib-0021] Loveday, C. , Turnbull, C. , Ruark, E. , Xicola, R. M. , Ramsay, E. , Hughes, D. ,… Rahman, N. (2012). Germline RAD51C mutations confer susceptibility to ovarian cancer. Nature Genetics, 44(5), 475–476; author reply 476. 10.1038/ng.2224 22538716

[mgg31070-bib-0022] Lu, H.‐M. , Li, S. , Black, M. H. , Lee, S. , Hoiness, R. , Wu, S. , … Elliott, A. (2018). Association of breast and ovarian cancers with predisposition genes identified by large‐scale sequencing. JAMA Oncology, 5(1), 51 10.1001/jamaoncol.2018.2956 PMC6439764 30128536

[mgg31070-bib-0023] Machado, P. M. , Brandao, R. D. , Cavaco, B. M. , Eugenio, J. , Bento, S. , Nave, M. , … Vaz, F. (2007). Screening for a BRCA2 rearrangement in high‐risk breast/ovarian cancer families: Evidence for a founder effect and analysis of the associated phenotypes. Journal of Clinical Oncology, 25(15), 2027–2034. 10.1200/JCO.2006.06.9443 17513806

[mgg31070-bib-0024] Mason, R. D. , Bowmer, M. I. , Howley, C. M. , & Grant, M. D. (2005). Cross‐reactive cytotoxic T lymphocytes against human immunodeficiency virus type 1 protease and gamma interferon‐inducible protein 30. Journal of Virology, 79(9), 5529–5536. 10.1128/JVI.79.9.5529-5536.2005 15827167 PMC1082750

[mgg31070-bib-0025] Meindl, A. , Hellebrand, H. , Wiek, C. , Erven, V. , Wappenschmidt, B. , Niederacher, D. , … Hanenberg, H. (2010). Germline mutations in breast and ovarian cancer pedigrees establish RAD51C as a human cancer susceptibility gene. Nature Genetics, 42(5), 410–414. 10.1038/ng.569 20400964

[mgg31070-bib-0026] Miller, S. A. , Dykes, D. D. , & Polesky, H. F. (1988). A simple salting out procedure for extracting DNA from human nucleated cells. Nucleic Acids Research, 16(3), 1215 10.1093/nar/16.3.1215 3344216 PMC334765

[mgg31070-bib-0027] Milting, H. , Klauke, B. , Christensen, A. H. , Musebeck, J. , Walhorn, V. , Grannemann, S. , … Anselmetti, D. (2015). The TMEM43 Newfoundland mutation p. S358L causing ARVC‐5 was imported from Europe and increases the stiffness of the cell nucleus. European Heart Journal, 36(14), 872–881. 10.1093/eurheartj/ehu077 24598986

[mgg31070-bib-0028] Mostafa, A. A. , Codner, D. , Hirasawa, K. , Komatsu, Y. , Young, M. N. , Steimle, V. , & Drover, S. (2014). Activation of ERalpha signaling differentially modulates IFN‐gamma induced HLA‐class II expression in breast cancer cells. PLoS ONE, 9(1), e87377 10.1371/journal.pone.0087377 24475282 PMC3903652

[mgg31070-bib-0029] Neidhardt, G. , Becker, A. , Hauke, J. , Horvath, J. , Bogdanova Markov, N. , Heilmann‐Heimbach, S. , … Hahnen, E. (2017). The RAD51C exonic splice‐site mutations c.404G>C and c.404G>T are associated with familial breast and ovarian cancer. European Journal of Cancer Prevention, 26(2), 165–169. 10.1097/CEJ.0000000000000240 27622768

[mgg31070-bib-0030] Nielsen, F. C. , van Overeem Hansen, T. , & Sorensen, C. S. (2016). Hereditary breast and ovarian cancer: New genes in confined pathways. Nature Reviews Cancer, 16(9), 599–612. 10.1038/nrc.2016.72 27515922

[mgg31070-bib-0031] Olufemi, S. E. , Green, J. S. , Manickam, P. , Guru, S. C. , Agarwal, S. K. , Kester, M. B. , … Chandrasekharappa, S. C. (1998). Common ancestral mutation in the MEN1 gene is likely responsible for the prolactinoma variant of MEN1 (MEN1Burin) in four kindreds from Newfoundland. Human Mutation, 11(4), 264–269. 10.1002/(SICI)1098-1004(1998)11:4<264:AID-HUMU2>3.0.CO;2-V 9554741

[mgg31070-bib-0032] Osorio, A. , Endt, D. , Fernández, F. , Eirich, K. , de la Hoya, M. , Schmutzler, R. , … Benitez, J. (2012). Predominance of pathogenic missense variants in the RAD51C gene occurring in breast and ovarian cancer families. Human Molecular Genetics, 21(13), 2889–2898. 10.1093/hmg/dds115 22451500

[mgg31070-bib-0033] Pang, Z. , Yao, L. U. , Zhang, J. , Ouyang, T. , Li, J. , Wang, T. , … Xie, Y. (2011). RAD51C germline mutations in Chinese women with familial breast cancer. Breast Cancer Research and Treatment, 129(3), 1019–1020. 10.1007/s10549-011-1574-3 21597919

[mgg31070-bib-0034] Pelttari, L. M. , Heikkinen, T. , Thompson, D. , Kallioniemi, A. , Schleutker, J. , Holli, K. , … Nevanlinna, H. (2011). RAD51C is a susceptibility gene for ovarian cancer. Human Molecular Genetics, 20(16), 3278–3288. 10.1093/hmg/ddr229 21616938

[mgg31070-bib-0035] Pelttari, L. M. , Nurminen, R. , Gylfe, A. , Aaltonen, L. A. , Schleutker, J. , & Nevanlinna, H. (2012). Screening of Finnish RAD51C founder mutations in prostate and colorectal cancer patients. BMC Cancer, 12, 552 10.1186/1471-2407-12-552 23176254 PMC3522023

[mgg31070-bib-0036] Pelttari, L. M. , Shimelis, H. , Toiminen, H. , Kvist, A. , Torngren, T. , Borg, A. , … Nevanlinna, H. (2018). Gene‐panel testing of breast and ovarian cancer patients identifies a recurrent RAD51C duplication. Clinical Genetics, 93(3), 595–602. 10.1111/cge.13123 28802053

[mgg31070-bib-0037] Petty, E. M. , Green, J. S. , Marx, S. J. , Taggart, R. T. , Farid, N. , & Bale, A. E. (1994). Mapping the gene for hereditary hyperparathyroidism and prolactinoma (MEN1Burin) to chromosome 11q: Evidence for a founder effect in patients from Newfoundland. American Journal of Human Genetics, 54(6), 1060–1066. 7911003 PMC1918205

[mgg31070-bib-0038] Richards, S. , Aziz, N. , Bale, S. , Bick, D. , Das, S. , Gastier‐Foster, J. , … Rehm, H. L. (2015). Standards and guidelines for the interpretation of sequence variants: A joint consensus recommendation of the American College of Medical Genetics and Genomics and the Association for Molecular Pathology. Genetics in Medicine, 17(5), 405–424. 10.1038/gim.2015.30 25741868 PMC4544753

[mgg31070-bib-0039] Romero, A. , Perez‐Segura, P. , Tosar, A. , Garcia‐Saenz, J. A. , Diaz‐Rubio, E. , Caldes, T. , & de la Hoya, M. (2011). A HRM‐based screening method detects RAD51C germ‐line deleterious mutations in Spanish breast and ovarian cancer families. Breast Cancer Research and Treatment, 129(3), 939–946. 10.1007/s10549-011-1543-x 21537932

[mgg31070-bib-0040] Sánchez‐Bermúdez, A. I. , Sarabia‐Meseguer, M. D. , García‐Aliaga, Á. , Marín‐Vera, M. , Macías‐Cerrolaza, J. A. , Henaréjos, P. S. , … Ruiz‐Espejo, F. (2018). Mutational analysis of RAD51C and RAD51D genes in hereditary breast and ovarian cancer families from Murcia (southeastern Spain). European Journal of Medical Genetics, 61(6), 355–361. 10.1016/j.ejmg.2018.01.015 29409816

[mgg31070-bib-0041] Schnurbein, G. , Hauke, J. , Wappenschmidt, B. , Weber‐Lassalle, N. , Engert, S. , Hellebrand, H. , … Hahnen, E. (2013). RAD51C deletion screening identifies a recurrent gross deletion in breast cancer and ovarian cancer families. Breast Cancer Research, 15(6), R120 10.1186/bcr3589 24359560 PMC3978715

[mgg31070-bib-0042] Shimelis, H. , LaDuca, H. , Hu, C. , Hart, S. N. , Na, J. , Thomas, A. , … Couch, F. J. (2018). Triple‐negative breast cancer risk genes identified by multigene hereditary cancer panel testing. Journal of the National Cancer Institute, 110(8), 855–862. 10.1093/jnci/djy106 30099541 PMC6093350

[mgg31070-bib-0043] Shirts, B. H. , Casadei, S. , Jacobson, A. L. , Lee, M. K. , Gulsuner, S. , Bennett, R. L. , … Pritchard, C. C. (2016). Improving performance of multigene panels for genomic analysis of cancer predisposition. Genetics in Medicine, 18(10), 974–981. 10.1038/gim.2015.212 26845104

[mgg31070-bib-0044] Song, H. , Dicks, E. , Ramus, S. J. , Tyrer, J. P. , Intermaggio, M. P. , Hayward, J. , … Pharoah, P. D. (2015). Contribution of germline mutations in the RAD51B, RAD51C, and RAD51D genes to ovarian cancer in the population. Journal of Clinical Oncology, 33(26), 2901–2907. 10.1200/JCO.2015.61.2408 26261251 PMC4554751

[mgg31070-bib-0045] Sopik, V. , Akbari, M. R. , & Narod, S. A. (2015). Genetic testing for RAD51C mutations: In the clinic and community. Clinical Genetics, 88(4), 303–312. 10.1111/cge.12548 25470109

[mgg31070-bib-0046] Sung, P.‐L. , Wen, K.‐C. , Chen, Y.‐J. , Chao, T.‐C. , Tsai, Y.‐F. , Tseng, L.‐M. , … Huang, C.‐Y. (2017). The frequency of cancer predisposition gene mutations in hereditary breast and ovarian cancer patients in Taiwan: From BRCA1/2 to multi‐gene panels. PLoS ONE, 12(9), e0185615 10.1371/journal.pone.0185615 28961279 PMC5621677

[mgg31070-bib-0047] Thompson, E. R. , Boyle, S. E. , Johnson, J. , Ryland, G. L. , Sawyer, S. , Choong, D. Y. H. , … Campbell, I. G. (2012). Analysis of RAD51C germline mutations in high‐risk breast and ovarian cancer families and ovarian cancer patients. Human Mutation, 33(1), 95–99. 10.1002/humu.21625 21990120

[mgg31070-bib-0048] Vuorela, M. , Pylkäs, K. , Hartikainen, J. M. , Sundfeldt, K. , Lindblom, A. , von Wachenfeldt Wäppling, A. , … Winqvist, R. (2011). Further evidence for the contribution of the RAD51C gene in hereditary breast and ovarian cancer susceptibility. Breast Cancer Research and Treatment, 130(3), 1003–1010. 10.1007/s10549-011-1677-x 21750962

[mgg31070-bib-0049] Yablonski‐Peretz, T. , Paluch‐Shimon, S. , Gutman, L. S. , Kaplan, Y. , Dvir, A. , Barnes‐Kedar, I. , … Friedman, E. (2016). Screening for germline mutations in breast/ovarian cancer susceptibility genes in high‐risk families in Israel. Breast Cancer Research and Treatment, 155(1), 133–138. 10.1007/s10549-015-3662-2 26687385

[mgg31070-bib-0050] Zannini, L. , Delia, D. , & Buscemi, G. (2014). CHK2 kinase in the DNA damage response and beyond. Journal of Molecular Cell Biology, 6(6), 442–457. 10.1093/jmcb/mju045 25404613 PMC4296918

[mgg31070-bib-0051] Zhai, G. , Zhou, J. , Woods, M. O. , Green, J. S. , Parfrey, P. , Rahman, P. , & Green, R. C. (2016). Genetic structure of the Newfoundland and Labrador population: Founder effects modulate variability. European Journal of Human Genetics, 24(7), 1063–1070. 10.1038/ejhg.2015.256 26669659 PMC5070906

