# Supplementary material for: A dominant RAD51C pathogenic splicing variant predisposes to breast and ovarian cancer in the Newfoundland population due to founder effect
Source: Mol Genet Genomic Med. 2019 Nov 28;8(2):e1070. doi: 10.1002/mgg3.1070 (PMC7005661; doi:10.1002/mgg3.1070)
Supplement: Supplementary file 1 [file MGG3-8-e1070-s001.docx]

# TITLE: A founder *RAD51C* pathogenic splicing variant predisposes to breast and ovarian cancer in the Newfoundland population

**Authors:** Lesa M. Dawson^1,2*^, Kerri N. Smith^1*^, Salem Werdyani^1^, Robyn Ndikumana^1^, Cindy Penney^1^, Louisa L. Wiede^1^, Kendra L. Smith^1^, Justin A. Pater^1^, Andrée MacMillan^2^, Jane Green^1^, Sheila Drover^1^, Terry-Lynn Young^1-3*^, Darren D. O’Rielly^1-3*^

**Affiliations:**

^1^Faculty of Medicine, Memorial University, St. John’s, Canada.

^2^Eastern Health Authority, St. John’s, Canada.

^3^Centre for Translational Genomics, St. John’s, Canada

*Contributed equally (shared first authorship and senior authorship)

# SUPPLEMENTARY MATERIAL

**Supplementary Methods**

***Whole-Exome Sequencing***

***Copy Number Variation (CNV) Analysis***

***Calculation of Variant Age***

***TOPO TA Cloning***

***Immunoblotting***

**Supplementary Table S1.** Newfoundland breast and ovarian cancer study criteria for HBOC clinical risk assignment.

**Supplementary Table S2.** Genes sequenced by commercial multigene panels.

**Supplementary Table S3.** Primer sequences used in this study designed using Primer3.

**Supplementary Results**

**Supplementary Table S4.** Whole-exome sequencing variants that passed filtering criteria.

**Supplementary Table S5.** *In* *silico* splicing analysis of the *RAD51C* c.571+4A>G variant.

**Supplementary Figure S1.** RAD51C (NP_478123; 42.2 kDA) protein expression in RIPA lysates from patient-derived BCL.

## Supplementary Methods

***Whole-Exome Sequencing***

The proband (PID: III-6) and her parents (PID: II-6 and II-7) of family 5440 were subjected to whole-exome sequencing to identify variants in candidate genes not examined by clinical multigene panel testing. Sequencing was performed at the Centre for Translational Genomics (CTG), a joint genomics facility operated by the Faculty of Medicine (Memorial University) and Eastern Health. High-quality genomic DNA (200 ng/sample) was amplified in Ion AmpliSeq Exome RDY plates using Ion AmpliSeq HiFi Mix (Ion Torrent). The resulting 240-280 bp products were treated with FuPa Reagent (Ion Torrent) to partially digest the primers and phosphorylate the amplicons, which were then ligated to Proton adapters and purified as per the manufacturer's instructions. Libraries were quantified by quantitative PCR and loaded onto the Ion Proton platform for high-throughput sequencing. The variant call format (VCF) files of the three samples were used for the functional annotation of genetic variants using ANNOVAR software (Wang, Li, & Hakonarson, 2010). Annotated variants were filtered, and the candidate variants were prioritized using an in-house analysis pipeline (CADD, SIFT, PolyPhen, Mutation Taster, LRT, PhyloP, GERP) using an allelic frequency of <1% (Lek et al., 2016). Amplicons with an average depth of coverage of at least 145X per amplicon (147.6, 154.3, 145.9) and at least 20X depth of coverage (97.46%, 97.04%, 96.60%) were generated from all samples tested.

***Copy Number Variation (CNV) Analysis***

The Agilent SurePrint G3 Human oligonucleotide CGH 1x1M Microarray kit (Agilent Technologies, #G4447-60510) was used to assess CNVs using the genomic DNA of the proband (PID: III-6) from family 5440. Briefly, 1.0 µg of the sample DNA and 1.0 µg of female human reference DNA (Agilent Technologies, #5190-3797) were digested and labelled using the SureTag Complete DNA Labelling Kit (Agilent Technologies, #5190-3399). The experimental and reference sample were labeled with Cyanine 5-dUTP and Cyanine 3-dUTP dyes, respectively. Subsequently, the experimental and reference samples were incubated in a thermocycler at 37 °C for 2 hours and 65 °C for 10 minutes. The labeled experimental and reference samples were then purified using purification columns (Agilent Technologies, #5190-3391). Yield and specific activity were determined using a Nanodrop 1000 and the experimental and reference sample were combined. The samples were then prepared for hybridization using the Oligo aCGH/ChIP-on-Chip Hybridization kit (Agilent Technologies, #5188-5220) and Human Cot-1 DNA (Invitrogen, # 15279-011), followed by incubation at 98 °C for 3 minutes and 37 °C for 30 minutes. The samples were immediately prepared for hybridization on the SurePrint G3 Human CGH Microarray and gasket slides (Agilent Technologies, #G2534-60003). The sample was hybridized in a 67 °C pre-warmed hybridization oven (Agilent Technologies, model G2545A) for 40 hours. The slide was washed using the Oligo aCGH/ChIP-on-chip Wash Buffer kit (Agilent Technologies, #5188-5221) and immediately placed in the slide holder, run on the Agilent Scanner D, and data extracted using Feature Extraction software. The resulting data was analyzed using Agilent CytoGenomics v4.0 software. A series of quality control analyses were performed to exclude intergenic CNVs and CNVs shorter than 10 Kbps in size. Perl programming language was used to find the overlap between the detected CNVs and previously identified and experimentally validated CNVs from the Database of Genomic Variants. Perl was also used to investigate the pathogenicity of the identified CNVs by inspecting the overlap between the CNVs in the experimental sample and CNVs in the ClinVar database, as well as in a dataset of CNVs observed in the NL population from the Medical Genetics Laboratory at Eastern Health (St. John’s, NL, CA).

***Calculation of Variant Age***

Generations (G) = logδ/log(1 – φ)

δ = (P_d_ – P_n_)/(1 – P_n_)

P_d_ = frequency of the ancestral microsatellite allele on the chromosome with the variant

P_n_ = frequency of the ancestral microsatellite allele on the chromosome without the variant

φ = recombination fraction (inferred from Halldorsson et al. 2019)

D17S1606:

φ = 1.981 cM x 0.01 = 0.0198

P_d_ = 14/17 = 0.8235

P_n_ = 6/17 = 0.3529

δ = (0.8235 – 0.3529)/(1 – 0.3529)

= 0.4706/0.6471

= 0.7272

G = log 0.7272/log(1 – 0.01981)

= -0.1383/-0.0087

= 15.897

15.897 gen. x 25 years = 397.4 years

D17S808:

φ = 2.612 cM x 0.01 = 0.0261

P_d_ = 12/17 = 0.7059

P_n_ = 11/17 = 0.6471

δ = (0.7059 – 0.6471)/(1 – 0.6471)

= 0.0588/0.3529

= 0.1666

G = log 0.1666/log(1 – 0.0261)

= -0.7783/-0.0115

= 67.68

67.68 gen. x 25 years = 1692.0 years

***TOPO TA Cloning***

*RAD51C* transcript splicing was investigated with TOPO TA cloning technology using the TOPO TA-Cloning Kit for Sequencing with One Shot TOP10 Chemically Competent E. coli (Invitrogen, #K457540) according to the manufacturer’s protocol. cDNA from patient-derived BCL was amplified with specific primers across *RAD51C* exons 2 and 7 (**Supplementary Table 3**). Clones were amplified using colony PCR (Costa & Weiner, 2006), sequenced on a 3130*xl* Genetic Analyzer (Applied Biosystems) and visualized using Mutation Surveyor Software v5.0.0 (SoftGenetics LLC).

***Immunoblotting***

Western blotting was conducted as described in the main manuscript using 10 or 20 µg of BCL-derived protein and 1 µg/mL polyclonal rabbit anti-RAD51C antibody (NP_478123.1: amino acids 1-130; Novus Biologicals, #NBP2-55278). Antibody binding was detected with 1:10,000 dilutions of horseradish peroxidase (HRP)-conjugated goat anti-mouse secondary antibody (Jackson Immunoresearch, #115-036-071) and Immobilon Western Chemiluminescent HRP Substrate (Millipore, #WBKLS0500). Immunoreactivity was visualized and quantified by scanning densitometry using an ImageQuant LAS 4000 and ImageQuant TL Software v8.1, respectively (GE Healthcare).

**Supplementary Table S1.** Newfoundland breast and ovarian cancer study criteria for HBOC clinical risk assignment.

| **Low Risk** | **Intermediate Risk** | **High Risk** |
| --- | --- | --- |
| • Ovarian cancer > 50 y  • No first- or second-degree  relatives with breast  cancer or other cancer  • Adequate family structure | • Any two of the following:  • Serous cell ovarian cancer < 65 y  • 2 first-degree relatives with  breast cancer or non-mucinous  epithelial ovarian cancer < 65 y  in two generations  • 1 first- or second-degree relative  with breast cancer < 50 y or  ovarian cancer < 60 y  • 1 first- or second-degree relative  with male breast cancer at any  age  • Bilateral breast cancer in one  individual  • Breast cancer and ovarian cancer  in one individual  • 2 second- or third-degree relatives  with breast cancer or ovarian  cancer < 65 y related via males  OR  • A pedigree that does not meet the  above criteria but demonstrates  second/third degree relatives with   breast cancer or ovarian cancer in a  less prominent pattern that does not  rule out HBOC by the judgement of  the team (e.g. due to limited  paternal structure, early non-cancer  deaths in female relatives, half sib-  ships, etc.) | • Any three of the following:  • Serous cell ovarian cancer <  65 y  • 2 first-degree relatives with  breast cancer or non-  mucinous epithelial ovarian  cancer < 65 y in 2 generations  • 1 first- or second-degree  relative with breast cancer <  50 y or ovarian cancer < 60 y |

**Supplementary Table S2.** Genes sequenced by commercial multigene panels.

| **Multigene panel** | **Genes sequenced** |
| --- | --- |
| GeneDx OncoGeneDx Custom Panel | *ATM*, *BARD1*, *BRCA1*, *BRCA2*, *BRIP1*, *CHD1*, *CHEK2*, *EPCAM*, *FANCC*, *MLH1*, *MSH2*, *MSH6*, NBN, PALB2, *PMS2*, *PTEN*, *RAD51C*, *RAD51D*, *TP53*, *XRCC2* |
| Invitae Common Hereditary Cancers Panel | *ATM*, *BARD1*, *BRCA1*, *BRCA2*, *BRIP1*, *CDH1*, *CHEK2*, *MRE11A*, *NBN*, *NF1*, *PALB2*, *PTEN*, *RAD50*, *RAD51C*, *STK11*, *TP53* |
| Fulgent Ovarian Cancer Focus Panel | *BARD1, BRCA1, BRCA2, BRIP1, EPCAM, MLH1, MRE11, MSH2, MSH6, NBN, PMS2, RAD51C, RAD51D, STK11, TP53* |

**Supplementary Table S3.** Primer sequences used in this study designed using Primer3.

| **Purpose** | **Target** | **Forward sequence** | **Reverse sequence** |
| --- | --- | --- | --- |
| Variant validation | *CHEK2* | ACCATATTCTGTAAGGACAGGACAA | AGCCCTCTGATGCATGCTTTTA |
|  | *RAD51C* | AGTTAGACATTTCTGTTGCCTTGGG | AGGAAGAAGTGAGGGCCATAAAAGG |
| Somatic sequencing | *RAD51C* | ATGTTTTGGAGGAGTGGCAG | TGGAGTGTTGCTGAGGTCTC |
| TaqMan genotyping | *RAD51C* | ATTCAGCACCTTCAGCTTATAGCA | AGATGGGCACAAATGCAAATTACTTT |
| Haplotyping | *D17S666* | CACTTTTGCCTTTTCTATGAGGTTT | CAACTGGAAATGGTATTCCCCAGC |
|  | *D17S1853* | GTCATTGTCGGGGGTG | TCAATCTATCTGTTTCTCTGTGTG |
|  | *D17S1606* | TGGTATTCAATCCTGGAGC | TGATGAGTCTTCATAGCCCC |
|  | *D17S1161* | GCCAAGATAATGCCATTGCA | TTCTCCCTGTGCCCTCTAA |
|  | *D17S1604* | CTGGGTGACAGAAACCTAAT | AGTACACAGCCAACATACACA |
|  | *D17S923* | ACCCAACATTGTAAATGGG | GCTGAGACCATATCACTTAAATC |
|  | *D17S808* | ACCCTAGACAGGATGCCA | TGTGGGTTTTCTCAGGTTAT |
|  | *D17S794* | GGCACAGTCTGCCACCTTTA | TGAGTTGCCACAGAGTGATG |
| RT-PCR cDNA analysis | *RAD51C* | GCACTGGAACTTCTTGAGCA | TCAAGAGTGAAATCCTCCAAAGC |
| TOPO TA cloning cDNA analysis | *RAD51C* | GCATACCCAGGGCTTCATAA | CTTTCGGTCCCAATGAAAGA |

## Supplementary Results

**Supplementary Table S4.** Whole-exome sequencing variants that passed filtering criteria.

| **Gene** | **Variant ID** | **Chromosome** | **Genomic Position** | **Allele** |
| --- | --- | --- | --- | --- |
| *FRMPD1* | rs140800271 | 9 | 37,733,752 | c.G1148A |
| *CACFD1* | rs146740814 | 9 | 136,330,532 | c.C283T |
| *DKK1* | rs149268042 | 10 | 54,074,798 | c.G359T |
| *RAB11FIP2* | rs150998302 | 10 | 119,798,924 | c.C824A |
| *KBTBD4* | rs770062216 | 11 | 47,594,609 | c.A1430G |
| *MTL5* | rs202023075 | 11 | 68,483,321 | c.G1004C |
| *CHRDL2* | rs144018357 | 11 | 74,424,490 | c.C230T |
| *TTC36* | rs118041771 | 11 | 118,399,392 | c.G193A |
| *CMKLR1* | rs141421422 | 12 | 108,686,555 | c.T185G |
| *DIAPH3* | rs200189161 | 13 | 60,384,960 | c.3125A |
| *PCK2* | rs111723834 | 14 | 24,572,932 | c.G1682A |
| *L3HYPDH* | rs201988078 | 14 | 59,950,623 | c.C412T |
| *GSE1* | rs750095056 | 16 | 85,667,696 | c.G184A |
| *AXIN2* | rs755990979 | 17 | 63,554,369 | c.G370A |
| *LAMA5* | rs144033127 | 20 | 60,910,176 | c.G2383A |
| *ACO2* | rs141878785 | 22 | 41,911,805 | c.G719C |

**Supplementary Table S5.** *In* *silico* splicing analysis of the *RAD51C* c.571+4A>G variant.

| **Program** | **Score** | |
| --- | --- | --- |
|  | **c.571+4A** | **c.571+4A>G** |
| MaxEntScan | 10.45 | 8.10 |
| GeneSplicer | 2.75 | - |
| NNSplice | 1.00 | 0.61 |
| SpliceSiteFinder | 90.73 | 80.65 |
| SplicePredictor | 0.987 | 0.946 |
| *FATHMM-XF* | - | 0.938 |


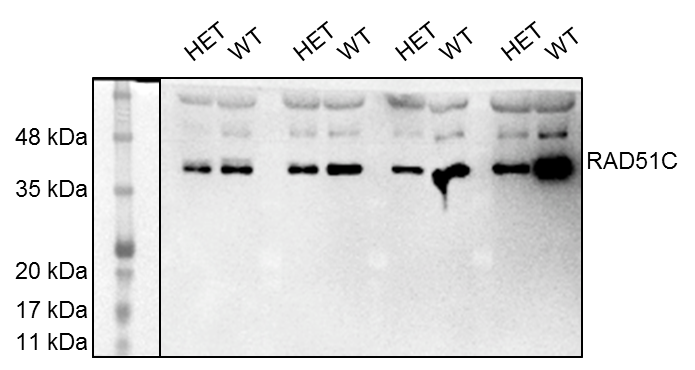


**Supplementary Figure S1.** RAD51C (NP_478123; 42.2 kDA) protein expression in RIPA lysates from patient-derived BCL. No small molecular weight bands corresponding to RAD51C isoform 2 (NP_002867; 14.9 kDA) or an alternate transcript produced due to exon 3 skipping (14.7 kDa) were observed following immunoblotting using a polyclonal anti-RAD51C antibody. Lane 1: ladder; lanes 2-9: 10 µg protein; lanes 11-12: 20 µg protein.

# REFERENCES

Costa, G. L., & Weiner, M. P. (2006). Colony PCR. *CSH Protoc, 2006*(1). doi:10.1101/pdb.prot4141

Halldorsson, B. V., Palsson, G., Stefansson, O. A., Jonsson, H., Hardarson, M. T., Eggertsson, H. P., . . . Stefansson, K. (2019). Characterizing mutagenic effects of recombination through a sequence-level genetic map. *Science, 363*(6425). doi:10.1126/science.aau1043

Lek, M., Karczewski, K. J., Minikel, E. V., Samocha, K. E., Banks, E., Fennell, T., . . . Exome Aggregation, C. (2016). Analysis of protein-coding genetic variation in 60,706 humans. *Nature, 536*(7616), 285-291. doi:10.1038/nature19057

Wang, K., Li, M., & Hakonarson, H. (2010). ANNOVAR: functional annotation of genetic variants from high-throughput sequencing data. *Nucleic Acids Res, 38*(16), e164. doi:10.1093/nar/gkq603
